# Deep Learning Models for FAMACHA©-Based Anemia Screening in Goats

**DOI:** 10.3390/ani16203301

**Published:** 2026-10-20

**Authors:** Mariline Hilaire, Hutama Arif Bramantyo, Sam Chamoun, Yin Sun, Honghe Wang, Santosh Chaudhary, Vivian Kanyi, Hossam Ismael, Heba Abdo, Byeng Ryel Min

**Affiliations:** 1 Department of Agricultural and Environmental Sciences, Tuskegee University, Tuskegee, AL 360088, USA; mhilaire6334@tuskegee.edu (M.H.); schaudhary8992@tuskegee.edu (S.C.); vkanyi1503@tuskegee.edu (V.K.); hismael@tuskegee.edu (H.I.); habdo@tuskegee.edu (H.A.); 2 Department of Electrical and Computer Engineering, Auburn University, Auburn, AL 36849, USA; hzb0071@auburn.edu (H.A.B.); samchamoun@auburn.edu (S.C.); yzs0078@auburn.edu (Y.S.); 3 Department of Biology, Tuskegee University, Tuskegee, AL 36849, USA; hwang@tuskegee.edu

**Keywords:** deep learning, FAMACHA©, goat anemia, image segmentation, ocular conjunctiva, vision transformer

## Abstract

*Haemonchus contortus* infection is a major cause of anemia in goats and can reduce productivity, reproductive performance, and animal welfare. The FAMACHA© system supports on-farm anemia screening by visually assessing ocular conjunctival color, but scoring can vary with evaluator experience, and lighting conditions. This study developed a smartphone-oriented deep learning framework for FAMACHA©-based anemia screening in goats using ocular conjunctival images. The dataset included 1995 FAMACHA©-labeled images from 141 meat and dairy goats collected across three acquisition campaigns. For the binary screening task, FAMACHA© scores of 4–5 were assigned to the anemic screening class, whereas scores of 1–3 were assigned to the non-anemic screening class. The pipeline combined U-Net-based conjunctiva segmentation with classification using six ImageNet-pretrained backbones: ResNet-50, ViT-B/16, Swin-T, EfficientNet-B0, MobileNetV4-Small, and MobileViT-v2-1.0. Models were evaluated using animal-level dataset splitting, nested cross-validation, validation-selected thresholds, goat-level cluster bootstrap confidence intervals and goat-block paired permutation tests. U-Net with a ResNet-34 encoder achieved a foreground IoU of 0.7342 and a Dice coefficient of 0.8372 for conjunctiva segmentation. On the held-out test set, ViT-B/16 achieved the highest anemia recall (0.924), accuracy (0.861), and anemia-class F1-score (0.879). However, MobileNetV4-Small performed closely, with 0.847 accuracy, a 0.861 F1-score, and the highest specificity (0.825). U-Net-ResNet34 combined with MobileNetV4-Small yielded the lowest estimated combined inference latency of 479.04 ms per image, calculated from separately measured segmentation and classification latencies on a Samsung SM-A556E smartphone using TensorFlow Lite. A Pearson’s correlation analysis showed a strong positive association between fecal egg count and FAMACHA© score (r = 0.7082, R^2^ = 0.5015, *p* < 0.0001). These results suggest that ocular-conjunctival imaging can support FAMACHA©-based anemia screening in goats, with ViT-B/16 serving as the strongest recall-oriented model and MobileNetV4-Small as a practical candidate for low-latency on-device inference under the tested smartphone configuration.

## Simple Summary

This study proposed a smartphone-based deep learning model for FAMACHA©-based anemia screening in goats using ocular conjunctival images. The model pipeline used a U-Net with a ResNet-34 encoder to segment the ocular conjunctival mucosa and classify images into FAMACHA©-based anemic and non-anemic screening categories, using six deep learning backbones spanning classical convolutional networks (ResNet-50, EfficientNet-B0), vision transformers, and mobile-oriented architectures (MobileNetV4-Small, MobileViT-v2-1.0). We studied and evaluated model consistency using intersection over union, Dice, precision, recall, accuracy, nested cross-validation, bootstrap confidence intervals, and goat-block paired permutation tests. The findings showed that ResNet-34 achieved the highest segmentation IoU and Dice coefficient among the evaluated U-Net encoders, while ViT-B/16 achieved the highest held-out classification accuracy and anemia-class F1-score. MobileNetV4-Small performed closely and provided the best practical accuracy–latency trade-off among the evaluated lightweight models, achieving the lowest estimated combined segmentation–classification inference latency at under 500 ms per image on the tested smartphone configuration.

## 1. Introduction

Anemia remains a prevalent issue in goat production, particularly in pasture-based and resource-limited agricultural settings [1]. If not diagnosed promptly, affected animals may experience weight loss, reduced reproductive success, and, in extreme cases, mortality. *Haemonchus contortus* is a primary cause of anemia in small ruminants; it is a hematophagous gastrointestinal parasite that lives in the abomasum and feeds on the host’s blood [1,2,3]. This parasite is challenging to manage in subtropical, tropical, and humid regions since these conditions promote its growth and persistence on pasture [4,5]. As anemia progresses, the mucous membrane often becomes lighter in color, ranging from red or pink in healthy animals to pale or nearly white in those that are severely affected [6]. The FAMACHA© system uses this observable change as a field screening tool. To assess anemia severity, veterinarians and trained farmers compare conjunctival color to a five-color scale to determine the need for anthelmintic treatment [6,7].

Two commonly used approaches for anemia assessment are packed cell volume (PCV), which provides a hematological measurement, and the FAMACHA© card, which provides a field-based visual screening assessment. However, these approaches have limitations. The PCV test involves laboratory assessment based on blood collection, measuring the percentages of white blood cells and red blood cells, and centrifugation, and requires trained workers. These stipulations make recurrent screening challenging on resource-constrained farms [8].

The FAMACHA© scoring method, while more user-friendly in field applications, still relies on subjective human judgment. Evaluator expertise, lighting, animal restlessness, and the clarity of lower-eyelid exposure can influence the score level [3,7,9]. This variability matters because an incorrect score can lead to two issues: an anemic animal may be overlooked, or a non-anemic animal may receive unnecessary treatment. Overlooked cases can harm animal welfare and productivity, whereas unwarranted treatment escalates anthelmintic drug resistance when using refugia-based parasite management [10].

Computer vision can enhance the objectivity and scalability of conjunctiva-based anemia screening in small ruminants. Previous research has shown that ocular conjunctiva images provide valuable data for FAMACHA© or anemia detection in small ruminants. Similarly, de Souza et al. [11] created a mobile FAMACHA© classifier for ovine species using a support vector machine trained on chromatic attributes, achieving 87% accuracy and 90% recall for anemia detection. In addition, Freitas et al. [3] employed random forest models and color-quantile characteristics to categorize five-level FAMACHA© scores in sheep, exhibiting significant performance variation across score levels. Freitas et al. [12] recently demonstrated that deep Convolutional Neural Network (CNN) models trained on sheep ocular images use PCV-derived anemia labels, with the optimal model achieving an F1-score of 0.62 for binary anemia classification. The findings demonstrate the viability of image-based screening; however, the same study showed that performance is strongly influenced by label definition, species, feature representation, and evaluation technique.

Nonetheless, numerous gaps persist, including the fact that most current image-based research has focused on sheep, while assessments relevant to goats remain scarce. This distinction matters, as goats may differ from sheep in ocular conjunctival appearance, management conditions, and FAMACHA© score levels. Also, image-level random splits with repeated photos of the same animal may cause identification leakage, producing overly positive assessments of generalization. Additionally, researchers have not evaluated contemporary transformer-based vision models and lightweight, mobile-oriented CNNs under a standardized animal-level protocol for this task. Statistical validation of model differences is often limited, complicating assessment of whether reported performance disparities are significant or merely due to sampling variability. Ultimately, realistic on-farm implementation requires considering both accuracy and inference delay on smartphone-grade hardware.

This study develops and evaluates a comprehensive deep learning system for FAMACHA©-based anemia screening in goats using ocular conjunctival images. The pipeline first segments the visible inner conjunctival area using a U-Net model with a ResNet-34 encoder, then classifies the cropped conjunctival image using six ImageNet-pretrained neural network architectures: ResNet-50, ViT-B/16, Swin-T, EfficientNet-B0, MobileNetV4-Small, and MobileViT-v2-1.0. We partition the dataset at the individual-goat level to ensure the same animal does not appear in more than one subset. We selected models and hyperparameters via nested cross-validation and assesd final performance on a reserved animal-level test set using goat-level cluster bootstrap confidence intervals and goat-block paired permutation tests. We additionally report smartphone-based segmentation and classification inference latency and their estimated combined latency to evaluate on-device model-inference feasibility.

The main contributions of this work are as follows: We curated a goat-specific FAMACHA©-labeled ocular conjunctiva image dataset containing 1995 images from 141 individual mixed goats across three acquisition campaigns. We developed a segmentation-based preprocessing pipeline to isolate the inner conjunctival mucosa before classification. We benchmarked six deep-learning backbones spanning CNN, transformer, hybrid transformer, and mobile-oriented architectures under a unified animal-level evaluation protocol. We evaluated model reliability using nested cross-validation, goat-level cluster bootstrap confidence intervals, and goat-block paired permutation tests on the held-out test set. We compared inference latency across the evaluated backbones on a specific smartphone configuration to assess on-device deployment feasibility under the tested hardware and runtime setup. This study aimed to develop an automated framework for FAMACHA©-based anemia screening from ocular conjunctival images by combining segmentation and classification in a unified pipeline.

The remainder of this paper is organized as follows. Section 2 reviews related work on image-based anemia screening and ocular conjunctival analysis. Section 3 describes the dataset, segmentation pipeline, classification framework, model selection procedure, evaluation metrics, statistical analysis, and latency benchmark. Section 4 presents the experimental results, Section 5 discusses the findings, and Section 6 concludes the paper with key findings, limitations, and future directions.

## 2. Related Work

Vision-based anemia screening using ocular conjunctiva images in small ruminants has mainly followed two directions: classical machine learning with handcrafted color features and deep learning with segmented or cropped conjunctiva images. Both directions highlight the potential of image-based screening, but they also leave methodological gaps. These gaps relate to animal-level dataset splitting, model comparison, statistical validation, and deployment feasibility evaluation. Early work treated FAMACHA© prediction largely as a color-feature classification problem. De Souza et al. [11] developed a smartphone-based system for sheep using a support vector machine trained on Red Green and Blue (RGB) color statistics extracted from the ocular conjunctiva. Their model achieved 87% accuracy and 90% anemia recall on a 317-image dataset, using FAMACHA© scores 3–5 as the positive class. Freitas et al. [3] studied a more difficult five-class FAMACHA© scoring task in sheep using random forest classifiers trained on color quantile features extracted after conjunctiva segmentation.

Model performance differed across score levels, with greater sensitivity in detecting FAMACHA© level 3 animals than in the more severe FAMACHA© 4 and 5 categories. These studies show that conjunctival color provides useful information for FAMACHA©-based anemia screening, but engineered features may limit the ability to capture more complex information related to mucosal texture, illumination variation, and image acquisition conditions. More recent studies have used deep learning to detect anemia through the ocular conjunctiva. Freitas et al. [12] used VGG19, Inception v3, and Xception models on 3441 images of sheep, using packed cell volume (PCV) rather than FAMACHA© score as the reference label. Using PCV < 27% as the definition of anemia, they achieved an F1-score of 0.62, an anemia recall of 0.83, and a precision of 0.49. Their pipeline also included U-Net-based segmentation of images derived from the Segment Anything Model with an IoU of 0.68 on test data.

This study demonstrates the value of deep learning and segmentation for ocular image analysis, but the use of PCV changes the practical setting: PCV provides a laboratory-based anemia reference, whereas FAMACHA© is the visual field screening tool commonly used in routine herd management.

Several gaps remain across these studies. First, animal-level dataset splitting is not always emphasized, even though repeated images from the same animal can significantly inflate test performance if the animal appears in both the training and test data subsets. Second, transformer-based models such as the Vision Transformer and the Swin Transformer have not been systematically compared with CNNs and lightweight, mobile-oriented backbones for goat FAMACHA© screening under the same evaluation protocol. Third, researchers often underreport uncertainty about model differences. Traditional paired statistical tests, such as McNemar’s test, can be used to compare classifier predictions, while cluster-aware procedures are more appropriate when repeated observations are obtained from the same animal. Finally, mobile inference costs are rarely evaluated, although smartphone deployment is central to practical use under field conditions.

Segmentation is also relevant to this task because the screening-relevant visual signal is concentrated in a small mucosal region. U-Net and encoder–decoder variants with ResNet backbones are widely used in biomedical and veterinary imaging to isolate regions of interest from limited annotations. In parallel, lightweight CNN families such as EfficientNet and MobileNetV4 are commonly considered for edge deployment because they offer favorable accuracy–latency trade-offs. These model families provide a useful basis for comparing high-capacity and deployment-oriented architectures in goat anemia screening.

On the basis of these gaps, the current research evaluates goat-specific FAMACHA©-based anemia screening using a unified pipeline that combines conjunctival segmentation, animal-level model evaluation, multiple backbone families, statistical validation, and mobile latency analysis. The goal is to identify the highest-performing model while also determining which neural network architecture provides a practical balance between FAMACHA©-based screening performance and computational cost.

## 3. Materials and Methods

### 3.1. Dataset Collection

The Tuskegee University Animal Care and Use Protocol Form approved all animal handling and image collection procedures under protocol numbers R08-2024-06 prior to collecting the images of goat conjunctiva. The dataset consisted of 1995 ocular conjunctiva images collected from 141 individual mixed-breed goats across three acquisition campaigns by one evaluator. Cohorts 1 and 2 were derived from an earlier longitudinal screening study, whereas Cohort 3 images were collected as a cross-sectional campaign for this study with more intensive per-animal sampling. Together, the three cohorts covered approximately 11 months of active data collection conducted in Tuskegee, Alabama (Macon County, United States of America (USA)) from two private farms and the Tuskegee University Caprine Research and Education Unit farm. This combined design captured both temporal variation from repeated weekly screening and inter-animal variation through cross-sectional sample collection.

Cohorts 1 and 2 included 690 images collected from 92 goats weekly between the summer of 2024 and summer 2025 across nine screening sessions. FAMACHA© scores were recorded at each morning visit, allowing the same animal’s score to change over time as its clinical condition changed. The cohort included all five FAMACHA© score levels, and, based on the majority score per animal, 13 of the 92 goats (14%) were classified as anemic, defined as a FAMACHA© score of 4 or 5.

The first cohort included forty-seven Spanish Boer goats aged 4–6 months that grazed on *Crotalaria juncea* L. (sunn hemp) and *Cynodon dactylon* (bermudagrass). The goats were allocated into 5 or 6 animals in half-acre plots having four replicates. The FAMACHA© score, and fecal egg count (FEC) of goats were recorded weekly for 102 days in summer 2024. Eight goats died from cohort 1 during the trial because of heavy *H. contortus* infection. The second cohort included forty-five Spanish Boer goats used during the summer of 2025 aged 5–7 months. Then goats were allocated to biochar treatments using low, medium, or higher biochar diets and fed a control. Ten goats per treatment were housed in individual pens and fifteen goats were assigned to the controls individually for a period of 90 days. FAMACHA© and FEC were collected weekly during the entire experimental period.

Cohort 3 included 1305 images from 49 crossbred meat and dairy goats, including males and females, collected over four weekly sessions in early April 2026. FAMACHA© scores and FEC were obtained from three farms on mixed-grass pasture. Fecal samples were collected from individual goats and placed into labeled Ziploc bags and put in a cooler filled with ice before being transported to the laboratory for analysis. Feces (1–4 g) were measured in our campus laboratory and analyzed using the Modified McMaster technique (Henricksen and Aagard, 1986 [13]).

Fecal egg counts and FAMACHA© scores were analyzed, including 133 goats across cohorts 1, 2, and 3, using one-way analysis of variance (ANOVA) with the General Linear Model (GLM) procedure of SAS software version 9.4 (SAS Institute Inc., Cary, NC, USA). One goat from farm 3 was empty for the FEC value. Therefore, 132 goats were used in the ANOVA, while 131 goats with complete FEC and FAMACHA© scores were used in the correlation analysis. The dependent variables used in this study were FAMACHA© scores and FEC (eggs per gram), which were analyzed separately. The treatment group was included as a fixed effect with nine levels (Bermudagrass, Sunn Hemp, Control, Low-, Medium-, and High-Biochar and farms 1–3). Cohorts 1 and 2 were measured for FEC and FAMACHA© repeatedly on the same goats weekly during each study period. The average value of individual goats was determined from all of their sampling days, which results in one average FEC and one average FAMACHA© score per each goat. For cohort 3, each goat provided only one FEC and one FAMACHA© reading. In summary, each individual goat contributed to one observation per variable. The least square means were estimated, and the differences among groups were assessed. The correlation between FAMACHA© scores and fecal egg counts (FEC) was evaluated using Pearson’s correlation coefficient (PROC CORR) in SAS based on 131 observations from goats. Statistical significance was set at *p* < 0.05.

Images were captured using a Samsung Galaxy Tab A9+ tablet with the built-in rear camera at 2032 × 3264 pixels. Cohort 3 also intentionally included more clinically anemic animals: 24 of the 49 goats (49%) had a majority FAMACHA© score of 4 or 5. Among the Cohort 3 images with polygon segmentation annotations, the FAMACHA© score distribution was as follows: 24 images at level 2, 463 at level 3, 483 at level 4, and 335 at level 5; this annotated subset contained no level 1 images. We explicitly reported this targeted sampling because the combined dataset was enriched for severe anemia cases and should not be interpreted as reflecting the natural herd-level prevalence of anemia.

**Figure 1:**
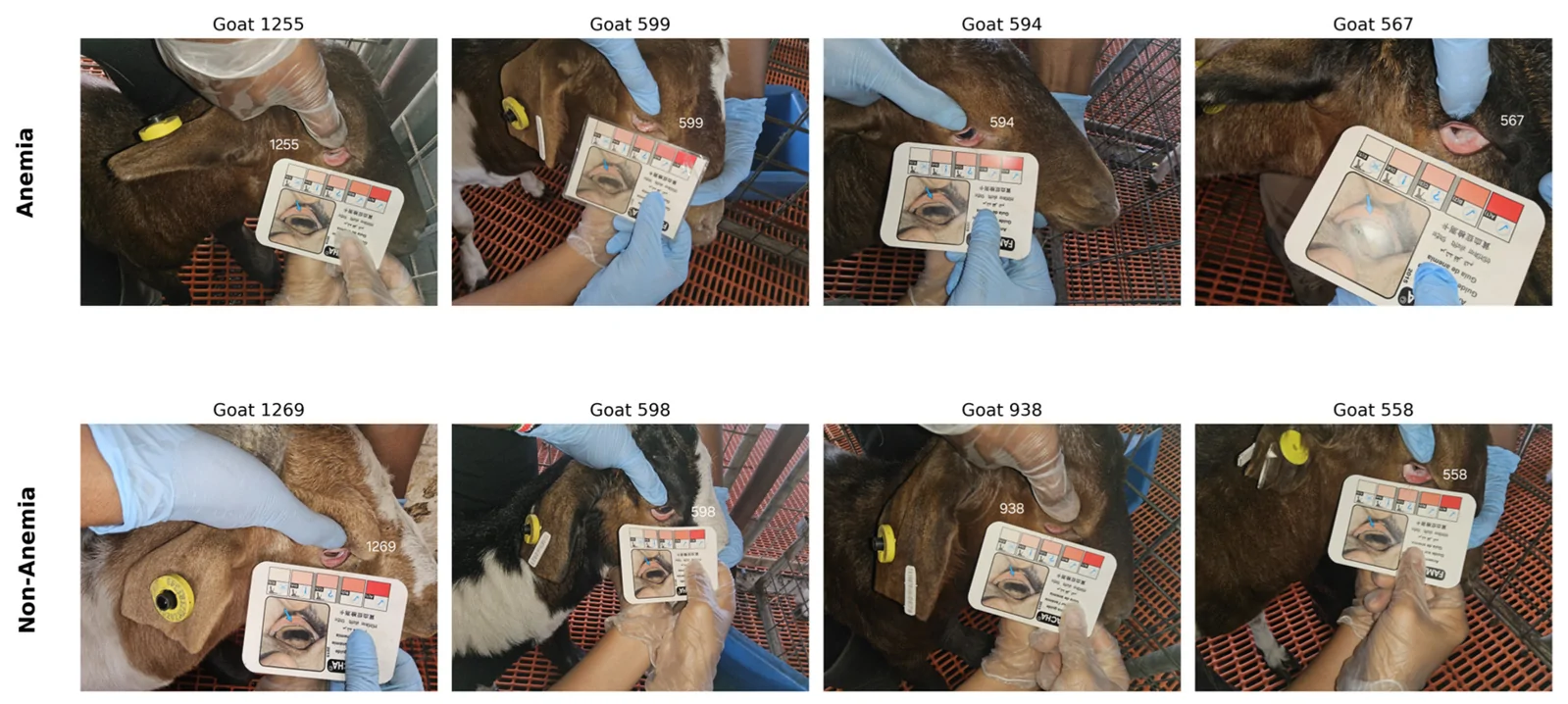
Field application of the FAMACHA© scoring protocol on study goats. The evaluator manually exposes the lower eyelid conjunctiva and compares its color with the FAMACHA© reference card to assign a score from 1 (deep red, healthy) to 5 (white, severely anemic). In each panel, the blue arrow indicates the example conjunctiva on the card, the number denotes the goat identifier, and the colored cells represent the FAMACHA© scale (1–5). The multilingual text on the card is part of the commercial FAMACHA© card, not an author annotation.

For each image, trained research personnel manually exposed the lower eyelid conjunctiva, and the camera was held approximately 15–30 cm from the eye. We acquired images under mixed field lighting conditions, including outdoor daylight and indoor barn lighting. At the time of imaging, one trained FAMACHA© evaluator assigned each image a FAMACHA© score from 1 to 5 following the chart protocol [6]. In this protocol, the exposed ocular conjunctiva is compared with a five-level reference card, where score 1 indicates deep-red, healthy mucosa and score 5 indicates pale or white mucosa associated with severe anemia (Figure 1).

For binary classification, images with FAMACHA© scores 4–5 were labeled as anemic, and images with scores 1–3 were labeled as non-anemic. This stricter definition focuses on severe anemia cases, where missed treatment is more clinically consequential, and differs from studies that classify FAMACHA© 3–5 as anemic [7,11]. Because goats could be photographed across multiple visits or sessions, we assigned labels at the image level rather than fixing them at the animal level.

### 3.2. Data Preparation

We split the classification dataset at the goat level rather than the image level to minimize identity leakage between training and evaluation. We assigned all images from the same goat to only one subset. We implemented the split using Stratified-GroupKFold [14], with goat identity as the group variable and the binary anemia label as the stratification target. We used a random seed of 42 to ensure reproducibility. This procedure generated 1150 training images from 84 goats, 341 validation images from 28 goats, and 504 test images from 29 goats (Table 1).

The combined dataset included 920 anemic images and 1075 non-anemic images, yielding an image-level anemia prevalence of 46.1%. The held-out test set dataset had 276 anemic and 228 non-anemic images, resulting in an anemic prevalence rate of 54.8%. As described in Section 3.1, this distribution reflects Cohort 3′s enrichment for clinically anemic animals and should not be interpreted as natural herd-level prevalence.

For segmentation model development, the 1305 images from 49 goats in Cohort 3 were manually annotated with polygon masks of the inner conjunctival mucosa and exported in COCO format [15] using Roboflow (accessed on 7 August 2024) [16]. Cohort 3 was selected for segmentation annotation because it was acquired under standardized tablet imaging and provided the largest, most consistent set of conjunctival images. Three trained annotators created the masks, with each image annotated by one annotator. The masks delineated only the visible inner conjunctiva and excluded eyelid skin, sclera, iris, and surrounding background.

The annotated Cohort 3 dataset was partitioned at the goat level using the same random seed and animal-level assignment strategy as the classification dataset, yielding 701 training, 231 validation, and 373 held-out test images from 12 goats. The Cohort 3 animal assignments were aligned across the segmentation and classification stages: the 12 goats in the segmentation test set were the same 12 Cohort 3 goats represented in the classification held-out test set. Therefore, no goat assigned to the held-out classification test set was used for segmentation training or validation-based encoder selection.

Two image versions were prepared. We used the original high-resolution images (2032 × 3264 pixels) for segmentation. For classification, we generated 224 × 224 conjunctival crops using the trained U-Net + ResNet-34 and segmented them. We used the predicted mask to compute a bounding box around the conjunctival region, then center-cropped with a small margin. We used these cropped images as input to the classification backbones described in Section 3.4.

### 3.3. Conjunctiva Segmentation

The visible inner conjunctiva represents the main image region used for FAMACHA©-based anemia assessment. We therefore trained U-Net segmentation models to separate this mucosal region from the surrounding eyelid, sclera, skin, FAMACHA© card, and background before classification. We evaluated three ResNet encoder backbones: ResNet-18, ResNet-34, and ResNet-50 [17,18]. We initialized the encoders with ImageNet-pretrained weights. We trained in two stages: first, we froze the encoder while the decoder learned the segmentation task, then fine-tuned the full network jointly. We resized input images to 512 × 512 pixels. A dropout layer [19] with a probability of 0.1 was applied between decoder blocks to reduce overfitting on the annotated subset.

We trained all three segmentation models under the same settings to enable a controlled comparison across encoder depths. We used the Adam optimizer with an initial learning rate of 1 × 10^−3^ and a batch size of 16. We used multiclass Dice loss [20] as the objective function, implemented in the Segmentation Models PyTorch library (version 0.5.0) with PyTorch 2.3.1, with two output classes: background and inner conjunctiva. We reduced the learning rate when validation loss plateaued, using a reduction factor of 0.5 and a patience of 5 epochs. We trained for up to 100 epochs, with early stopping if validation loss did not improve for 15 consecutive epochs.

At inference, we obtained the predicted segmentation mask by taking the argmax over the two output channels. We postprocessed the resulting binary conjunctiva mask with a 3 × 3 morphological opening followed by a 3 × 3 morphological closing. This step removed small isolated false-positive regions and smoothed minor boundary irregularities. We used the postprocessed masks for segmentation evaluation and to extract the conjunctival crops used in the classification stage.

The three encoder backbones were evaluated on the same animal-level split described in Section 3.2. For each encoder, the checkpoint with the highest validation Dice coefficient was retained. Encoder selection for the downstream classification pipeline was based solely on validation Dice performance. ResNet-34 achieved the highest validation Dice coefficient (0.8200), followed by ResNet-50 (0.8177) and ResNet-18 (0.8162). ResNet-34 was therefore selected to generate the 224 × 224 conjunctival crops used for classification. The held-out segmentation test set was used only for final performance evaluation, as reported in Section 4.1.

### 3.4. FAMACHA©-Based Classification

We evaluated six ImageNet-pretrained backbone architectures for binary FAMACHA©-based classification: ResNet-50 [18], ViT-B/16 [21], Swin-T [22], EfficientNet-B0 [23], MobileNetV4-Small [24], and MobileViT-v2-1.0 [25]. We selected these models to represent conventional CNNs, transformer neural networks, and lightweight neural networks for mobile devices, each with distinct accuracy and efficiency profiles. For each backbone, we replaced the original classifier with a task-specific linear head that produces two logits for the non-anemic and anemic classes.

Because missed anemic cases are clinically more concerning than false positives in a screening setting, we trained the model with focal loss [26] with γ = 2.0. Class-balanced α weights were computed from the training-set class distribution, giving approximately 1.10 for the anemic class and 0.90 for the non-anemic class.

We trained all models using AdamW [27]. Training followed a transfer-learning schedule with a one-epoch head warm-up, during which we froze the backbone, followed by full-network fine-tuning. A two-epoch linear learning-rate warm-up was applied before cosine decay over the remaining epochs. Models were trained for up to 30 epochs, with early stopping allowed only after epoch 12 to avoid stopping larger backbones before convergence. Depending on the selected configuration, models use either discriminative learning rates, with a higher learning rate for the classifier head than the backbone, or a uniform learning rate across all trainable layers.

During training, we augmented images using RandAugment [28], followed by random horizontal flipping with probability 0.5. We normalized pixel values using the ImageNet mean and standard deviation. Validation and test images were not augmented and received only the same normalization step. We selected additional regularization parameters, including dropout rate and stochastic depth/drop-path rate [29], during hyperparameter tuning, which is supported by the backbone implementation. The final hyperparameters for each model were selected using the nested cross-validation procedure described in Section 3.5.

### 3.5. Hyperparameter Selection via Nested Cross-Validation

We selected hyperparameters using nested cross-validation on the combined training and validation set. Both the inner and outer folds were stratified by the binary anemia label and grouped by the goat identifier, so that no goat appeared in both training and evaluation partitions within the same fold. The outer loop used five folds to assess model-selection stability, and the inner loop used three folds for hyperparameter tuning. This animal-level nested design was employed to reduce identity leakage but ensure that the held-out test set was reserved until the final evaluation step [30,31].

For ResNet-50, ViT-B/16, and Swin-T, we used a two-stage inner search. We trained the classification heads exclusively in the first step, while freezing the backbone, and varied the head learning rate, weight decay, and label smoothing. In the second step, we unfroze the full network and refined the backbone learning rate, MixUp strength [32], dropout rate, and drop-path rate. In contrast, the mobile-oriented backbones, namely EfficientNet-B0, MobileNetV4-Small, and MobileViT-v2-1.0, were tested; the search also considered configurations with uniform and discriminative learning rates, because their training behavior differed across pilot runs. Table 2 summarizes the hyperparameter search space.

We selected the best configuration within each outer fold based on inner-fold performance. After completing all five outer folds, we used the most consistently selected configuration for each backbone to train the final model. This choice reduced dependence on a single fold-specific optimum. Final training used the training split, while we retained the validation split for early stopping and threshold selection.

For each classification model, the operating threshold was selected on the validation set by maximizing the anemia-class F1-score over candidate thresholds from 0.05 to 0.95 in increments of 0.05. The selected thresholds were 0.40 for ResNet-50, 0.70 for ViT-B/16, 0.50 for Swin-T, 0.50 for EfficientNet-B0, 0.45 for MobileNetV4-Small, and 0.35 for MobileViT-v2-1.0. Each threshold was then fixed and applied without modification to the held-out test set. No formal probability-calibration procedure was performed. Table 3 reports the final selected hyperparameters and validation-selected thresholds.

### 3.6. Statistical Analysis

We used two statistical procedures to assess the reliability of model performance on the held-out test set while accounting for repeated images from the same animal. First, goat-level cluster bootstrap confidence intervals were computed for individual model metrics. Second, goat-block paired permutation tests were used for pairwise model comparison.

For bootstrap analysis, the 29 goats in the held-out test images were resampled with replacement 4000 times. Whenever a goat was selected, all test images belonging to that goat were retained together in the bootstrap sample, thereby preserving the within-goat dependence among repeated images. For each bootstrap sample, we recomputed accuracy, the anemia-class F1-score, the anemia recall, the anemia precision, the specificity, and the balanced accuracy. The 95% confidence intervals were calculated using the percentile method, with the lower and upper bounds defined by the 2.5th and 97.5th percentiles of the bootstrap distributions [33]. The reported point estimates remained image-level performance measures, whereas the confidence intervals accounted for clustering at the individual-goat level.

For pairwise model comparison, traditional paired statistical tests such as McNemar’s test have been used to compare classifier performance on the same set of observations [34,35]. However, because the held-out test set in this study contained repeated images from the same goats, we used a goat-block paired permutation test to account for within-goat dependence. For each pair of models, the observed test statistic was the difference in overall image-level accuracy between the two models. Under the null hypothesis of equivalent predictive performance, the predictions of the two models were exchanged as a block within each goat with probability 0.5. Thus, all images belonging to the same goat were kept together during each permutation, preserving the within-goat dependence structure. The null distribution was estimated using 100,000 permutations. Two-sided *p*-values were calculated as the proportion of permuted absolute accuracy differences at least as large as the observed absolute difference, using the standard Monte Carlo correction. Because six backbones produced 15 pairwise comparisons, the raw *p*-values were adjusted using Holm’s step-down procedure [36] to control the family-wise error rate. Both raw and Holm-adjusted *p*-values were reported, and statistical significance was assessed using the adjusted *p*-values at α = 0.05.

### 3.7. Evaluation Metrics

For the classification task, we reported accuracy, anemia recall, specificity, anemia precision, balanced accuracy, and anemia-class F1-score. Balanced accuracy was calculated as the average of anemia recall and specificity, providing a measure that gives equal importance to both classes. We used anemia recall as the primary clinical metric because the intended screening use case prioritizes reducing missed anemic animals. We also reported specificity and precision alongside recall to show the trade-off introduced by the validation-selected thresholds. We used the anemia-class F1-score as a summary measure of the precision–recall balance at each model’s selected operating point.

For the segmentation task, we reported pixel accuracy, foreground intersection-over-union (IoU), foreground Dice coefficient, and macro F1-score across the background and conjunctiva classes. Foreground IoU and Dice were treated as the primary segmentation metrics because the conjunctival mucosa occupies only a small fraction of each full-resolution image. We reported pixel accuracy for completeness, but it is dominated by the background class and can remain high even when foreground localization is imperfect.

As a secondary generalizability analysis, held-out test predictions were also stratified by acquisition cohort and evaluated using the same validation-selected operating thresholds, without retraining or threshold re-selection.

### 3.8. On-Device Latency Benchmark

To assess on-device inference feasibility, we benchmarked the segmentation and classification stages separately on the tested smartphone configuration. Each trained classification backbone was exported from PyTorch to ONNX and converted to TensorFlow Lite (TFLite). Classification models were evaluated at FP32 precision using 224 × 224 cropped conjunctiva inputs, whereas the U-Net segmentation models were evaluated at FP32 precision using 512 × 512 inputs, matching the segmentation training resolution.

On-device inference was evaluated on a Samsung SM-A556E smartphone running Android 16 using TensorFlow Lite. Latency was measured using the TensorFlow Lite Benchmark APK with four inference threads, five warm-up runs, and 30 inference runs for each model configuration. Latency was reported as mean ± standard deviation. These measurements represent inference performance under the specific device and runtime configuration evaluated in this study and should not be interpreted as general performance across smartphone platforms.

For the combined two-stage latency analysis, segmentation and classification inference times were measured separately on the same smartphone configuration. The reported combined latency was estimated as the sum of the mean U-Net segmentation latency and the mean classification latency for each backbone. Image resizing, mask post-processing, bounding-box computation and cropping, image normalization, animal handling, image capture, data transfer, and user-interface processing were not included in this estimate. Therefore, the reported combined latency should not be interpreted as a measured runtime of the complete application.

### 3.9. Integrated Screening Pipeline

The complete inference pipeline connects the segmentation and classification stages sequentially. First, a raw ocular image was resized to 512 × 512 pixels and passed through the selected U-Net + ResNet-34 segmentation model. The predicted conjunctival mask was then postprocessed using the morphological opening and closing operations described in Section 3.3. A bounding box was computed from the resulting conjunctival region, and the corresponding region was cropped from the original image with a small margin. The crop was then resized to 224 × 224 pixels and normalized using the ImageNet mean and standard deviation before being passed to the selected classification backbone.

The classifier produces a probability for the anemic class. This probability was compared with the model-specific operating threshold selected on the validation set, as reported in Table 3. Images with probabilities equal to or above the selected threshold are assigned to the FAMACHA©-based anemic screening class, whereas those below the threshold are assigned to the non-anemic screening class. Thus, the full inference sequence is raw ocular image → conjunctiva segmentation → mask postprocessing → conjunctival crop → image normalization → FAMACHA©-based classification → threshold-based screening output.

For the mobile latency analysis, the segmentation and classification modules were benchmarked separately on the same Samsung SM-A556E device. The reported combined two-stage inference latency was estimated by summing the mean latency of the two modules. The preprocessing and intermediate operations connecting the two stages, including resizing, mask post-processing, cropping, and normalization, were not included in the latency estimate. Therefore, these results should not be interpreted as measurements of the complete application runtime. A prototype mobile application has been developed to integrate the full workflow, but its complete runtime was not benchmarked in the present study.

## 4. Results

### 4.1. Segmentation Results

**Figure 2:**
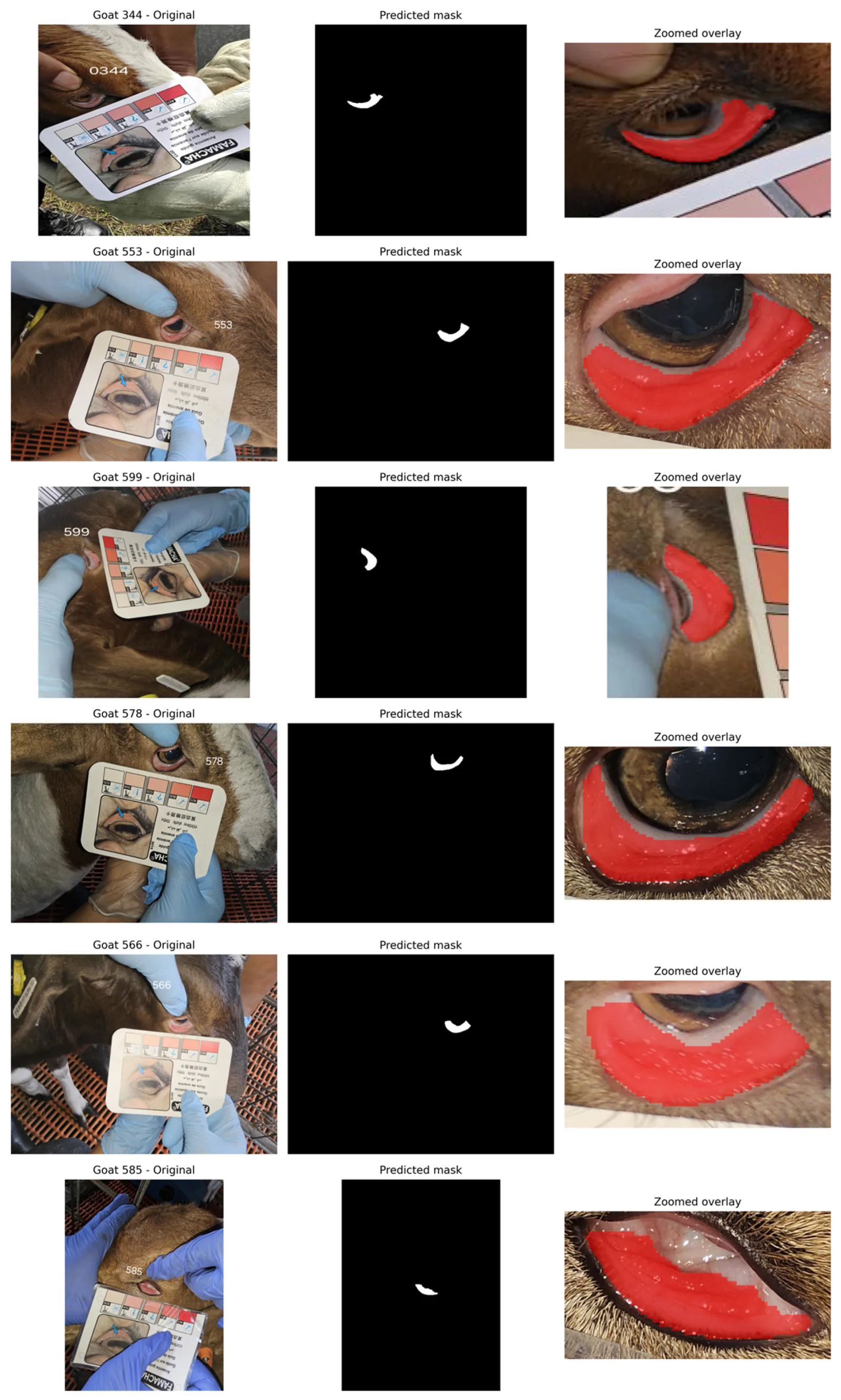
Representative examples of conjunctival mucosa segmentation. Each row shows the original field image, the predicted binary mask, and a zoomed overlay of the segmented conjunctival region. The number in each original image denotes the individual goat identifier, and the red region in the overlay indicates the conjunctival mucosa segmented by the model.

Figure 2 provided representative qualitative examples of the conjunctiva segmentation results. Each row showed the original field image, the predicted binary mask, and a zoomed overlay of the segmented region. Across variations in image orientation, animal appearance, and conjunctival exposure, the predicted masks generally localized the visible inner conjunctival region while excluding most surrounding background. The conjunctival region occupied only a small fraction of the image pixels, making foreground-based metrics more informative than overall pixel accuracy. Table 4 summarizes the corresponding test-set segmentation performance.

All three encoders achieved similar performance, with foreground IoU above 0.72 and Dice coefficient above 0.83. U-Net with a ResNet-34 encoder produced the highest overlap scores, with an IoU of 0.7342 and a Dice coefficient of 0.8372. ResNet-18 and ResNet-50 performed only slightly worse, indicating that changing encoder depth did not yield substantial gains on this dataset.

The primary distinction among encoders was the precision-recall trade-off. ResNet-50 achieved the best foreground precision (0.8739) but a lower recall (0.8204), indicating more cautious mask predictions. Conversely, ResNet-18 and ResNet-34 yielded more equitable precision and recall, both approximately 0.84–0.85. Pixel accuracy exceeded 0.999 for all models because the background class dominated the image area, confirming that IoU and Dice are more informative segmentation metrics in this context.

As described in Section 3.3, U-Net with a ResNet-34 encoder was selected for downstream crop generation based on the highest validation Dice coefficient. On the held-out segmentation test set, ResNet-34 also achieved the highest foreground IoU (0.7342) and Dice coefficient (0.8372). Previous studies on sheep ocular conjunctiva segmentation reported an average IoU of 0.70 ± 0.08 using a random-forest-based segmentation method [3] and a test-set IoU of 0.68 using U-Net-based segmentation [12]. The ResNet-34 model in the present study achieved a test IoU of 0.7342; however, direct comparisons should be interpreted cautiously because of differences in species, annotation procedures, and segmentation methods.

### 4.2. Hyperparameter Selection: Nested Cross-Validation Results

Following the nested cross-validation procedure described in Section 3.5, we report outer-fold accuracy and an anemia F1-score in Table 5, with a per-fold breakdown in Table 6. Table 3 lists the hyperparameter configurations selected through this process and used for final retraining. Swin-T achieved the highest mean outer-fold accuracy at 86.92 ± 5.05%, followed closely by MobileViT-v2-1.0 (86.52 ± 8.42%), ResNet-50 (86.26 ± 4.58%), and MobileNetV4-Small (86.18 ± 8.64%). EfficientNet-B0 reached 84.74 ± 9.09%, while ViT-B/16 had the lowest mean outer-fold accuracy at 83.23 ± 5.65%.

Between-fold variability was quite large. The standard deviation ranged from 4.6 to 9.1 percentage points, while the mean accuracy gap among the top four models was less than 1 percentage point. This means accuracy alone is not enough to rank the models reliably under animal-level cross-validation. A likely reason is the small number of goats in each outer fold, about 28, along with differences in appearance, image quality, and anemia severity within each fold. Related FAMACHA© image-analysis studies [3] have also reported similar drops in apparent performance under stricter validation settings, such as leaving out entire farms.

The F1-scores showed the same overall pattern. Swin-T had the highest mean F1 (84.86 ± 5.41%), followed closely by ResNet-50 (84.29 ± 4.74%). MobileViT-v2-1.0, MobileNetV4-Small, and EfficientNet-B0 were also close, all around 83%. ViT-B/16 had the lowest mean F1 at 78.58 ± 8.35%. Because the standard deviations are fairly large, the held-out test set in Section 4.3 is still needed to rank the models clearly.

The per-fold results in Table 6 show that some lightweight models were less stable than ResNet-50. MobileViT-v2-1.0 and MobileNetV4-Small performed very well on their best folds, with accuracy above 93%, but dropped to about 76 to 80% on their weakest folds. ResNet-50 varied less across folds, which supports its role as a stable reference model. Swin-T had the best mean performance in nested cross-validation, but the operating thresholds were selected using the validation set, while final model performance was assessed on the held-out test set in Section 4.3.

### 4.3. Held-Out Test Set Classification Results

**Figure 3:**
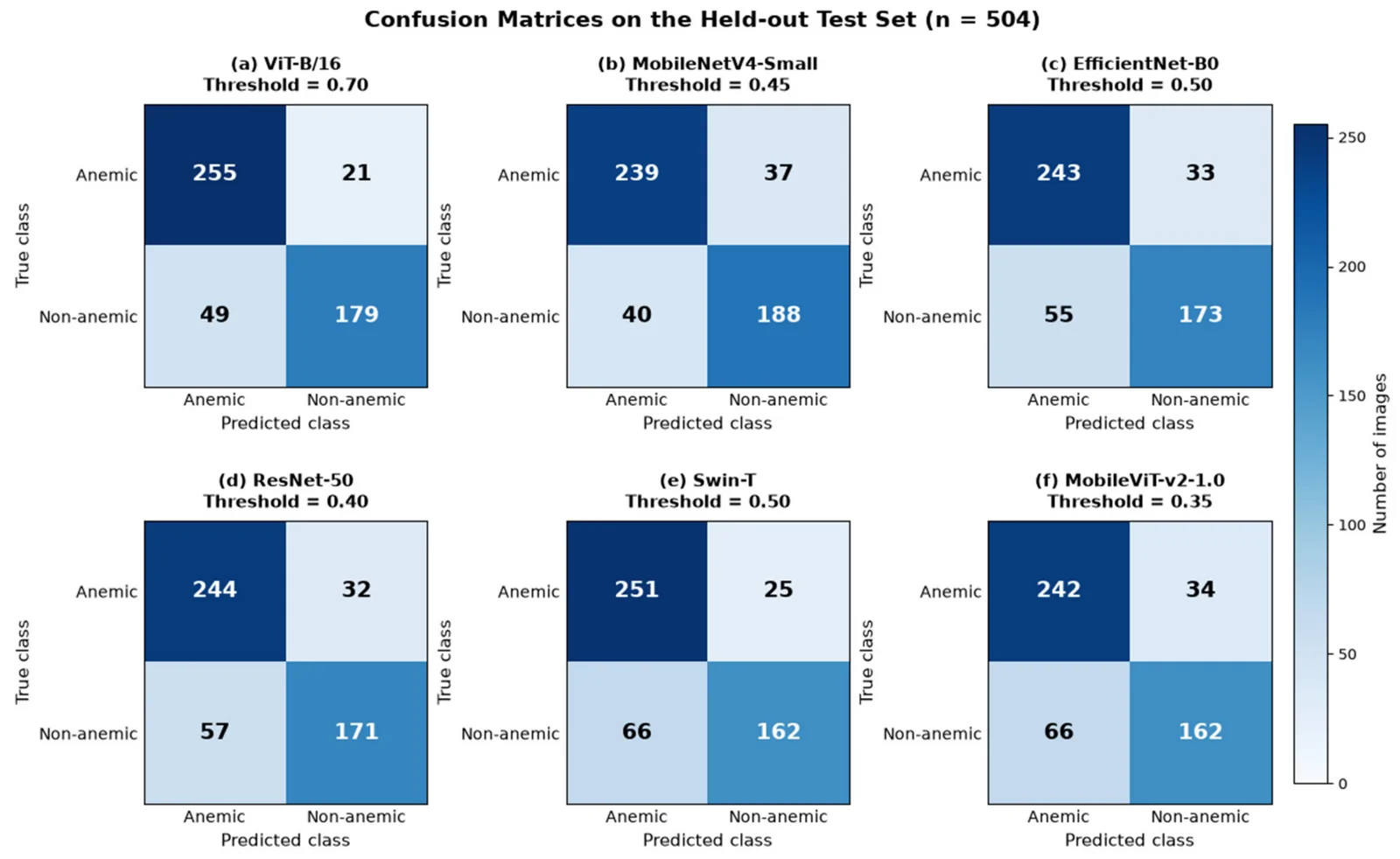
Confusion matrices for the six deep-learning classifiers on the held-out test set (*n* = 504 images from 29 goats). Rows represent the true class and columns represent the predicted class. (**a**) ViT-B/16, (**b**) MobileNetV4-Small, (**c**) EfficientNet-B0, (**d**) ResNet-50, (**e**) Swin-T, and (**f**) MobileViT-v2-1.0. Each model was evaluated using its validation-selected operating threshold.

After hyperparameter selection, we trained each architecture with its selected configuration and evaluated it on the held-out test set of 504 images from 29 goats. Each model used the validation selected operating threshold described in Section 3.5. Table 7 summarizes the classification results, and Figure 3 showed the corresponding confusion matrices.

ViT-B/16 achieved the strongest held-out test performance, with an accuracy of 0.861 [0.779, 0.921], anemia-class F1-score of 0.879 [0.697, 0.941], and anemia recall of 0.924 [0.753, 0.985]. MobileNetV4-Small followed closely, reaching an accuracy of 0.847 [0.747, 0.908] and an anemia-class F1-score of 0.861 [0.642, 0.928]. EfficientNet-B0 and ResNet-50 produced similar results, with accuracies of 0.825 [0.722, 0.890] and 0.823 [0.729, 0.887], respectively. Swin-T achieved an accuracy of 0.819 [0.705, 0.899] and the second-highest anemia recall, 0.909 [0.679, 0.987]. MobileViT-v2-1.0 had the lowest held-out accuracy among the six models, at 0.802 [0.680, 0.884], but still maintained an anemia recall of 0.877 [0.614, 0.967]. Balanced accuracy followed a similar pattern, with ViT-B/16 achieving the highest value (0.855), followed by MobileNetV4-Small (0.845).

Across all six backbones, anemia recall exceeded 0.86, ranging from 0.866 for MobileNetV4-Small to 0.924 for ViT-B/16. The validation-selected thresholds therefore maintained high sensitivity to the FAMACHA©-based anemic class while balancing precision and recall through the validation F1-score criterion. In a screening context, false-positive predictions may prompt additional assessment, whereas false-negative predictions may result in an anemic animal being missed during screening.

The confusion matrices in Figure 3 showed the same sensitivity-specificity trade-off more directly. ViT-B/16 missed 21 of 276 anemic images and produced 49 false positives among 228 non-anemic images. Swin-T also showed high sensitivity, missing 25 of 276 anemic images, but it produced 66 false positives. MobileNetV4-Small had the highest specificity among the tested models, correctly classifying 188 of 228 non-anemic images, while still detecting 239 of 276 anemic images. This makes MobileNetV4-Small a strong practical candidate, as reducing unnecessary follow-up checks is also important. ViT-B/16 remained the strongest overall model because it combined the highest anemia recall with the highest accuracy and F1-score.

To examine performance across acquisition conditions, we also stratified the held-out test set by cohort (Table 8). Cohorts 1&2 contained 131 images from 17 goats, whereas Cohort 3 contained 373 images from 12 goats. ViT-B/16 achieved the highest balanced accuracy in both groups, with 0.804 in Cohorts 1&2 and 0.838 in Cohort 3. Its corresponding accuracy and anemia recall were 0.824 and 0.773 in Cohorts 1&2, compared with 0.874 and 0.937 in Cohort 3. Similar cohort-related variation was observed across the other backbones. These differences should be interpreted cautiously because the two groups differed substantially in class composition, with an anemia prevalence of 16.8% in Cohorts 1&2 and 68.1% in Cohort 3. Therefore, the observed differences may reflect both acquisition-related conditions and differences in cohort composition.

### 4.4. FEC and FAMACHA©

A one-way analysis of variance (ANOVA) was performed to evaluate differences in FAMACHA© scores and FEC among the nine experimental groups. Our finding of the experimental group significantly affected FAMACHA© scores (*p* < 0.0001, R^2^ = 0.4696), explaining 46.96% of the variation. Likewise, the experimental group significantly affected fecal egg count (FEC) scores (*p* < 0.0001, R^2^ = 0.3634), explaining 36.34% of the variation. A Pearson’s correlation analysis was performed, and the result showed a strong positive association between FEC and FAMACHA© scores (r = 0.7082, R^2^ = 0.5015, *p* < 0.0001), indicating that about 50.15% of the variance of the FAMACHA© scores was in common with FEC. Goats showed more conjunctival paleness as *H. contortus* increased, as seen by rising fecal egg counts.

### 4.5. Statistical Validation

To assess whether the observed differences among the six classification models remained statistically meaningful after accounting for repeated images from the same animal, we performed pairwise goat-block permutation tests on the held-out test set. For each model pair, the observed statistic was the difference in overall image-level accuracy, while predictions were exchanged between the two models as a block within each goat during permutation. This procedure preserves the within-goat dependence among repeated images.

Table 9 summarizes the 15 pairwise comparisons using both raw and Holm-adjusted *p*-values. After adjustment for multiple comparisons, only the difference between ViT-B/16 and MobileViT-v2-1.0 remained statistically significant (accuracy difference = 0.0595, raw *p* = 0.0017, Holm-adjusted *p* = 0.0255). The comparison between ViT-B/16 and Swin-T was nominally significant before adjustment (raw *p* = 0.0347) but was not significant after Holm correction (adjusted *p* = 0.4858). Similarly, the comparison between MobileNetV4-Small and MobileViT-v2-1.0 had a raw *p*-value of 0.0486 but was not significant after adjustment (adjusted *p* = 0.6318). The remaining pairwise comparisons were also not statistically significant after the Holm correction.

Overall, the adjusted analysis indicates that most observed differences in held-out accuracy were not statistically distinguishable after controlling for multiple comparisons. Although ViT-B/16 had the highest held-out point accuracy, statistical evidence of superior accuracy was retained only in comparison with MobileViT-v2-1.0.

## 5. Discussion

### 5.1. Comparison with Prior Work

Table 10 compared the current findings with three pertinent studies on image-based anemia screening in small ruminants. Variations in species, label definitions, dataset sizes, model types, and evaluation protocols across these studies mean comparisons should be viewed contextually rather than as direct rankings.

The nearest mobile-focused comparison was de Souza et al. [11], who created a sheep FAMACHA© classifier utilizing an SVM with RGB color attributes. Their binary task characterized anemia using FAMACHA© scores of 3–5, achieving an F1-score of 0.88 and a recall of 0.90 on 317 sheep photos. In contrast, our top-performing model, ViT-B/16, achieved an anemia F1-score of 0.879 and an anemia recall of 0.924 on the held-out test set, using a goat-specific dataset comprising 1995 images and a stricter anemia definition of FAMACHA© scores 4–5. MobileNetV4-Small performed competitively, achieving an F1-score of 0.861 and a recall of 0.866, while showing higher specificity than ViT-B/16. These results indicated that the proposed goat-specific pipeline achieved performance comparable to previous mobile FAMACHA©-based work while targeting more severe FAMACHA© categories.

Freitas et al. [3] investigated five-class FAMACHA© prediction in sheep with random forest models and color-quantile characteristics. Their reported sensitivity varied across FAMACHA© levels, with better performance for score 3 than for scores 4 and 5. Because our work was binary rather than five-class, the findings were not directly comparable. This contrast suggests a practical advantage of using a binary screening target focused on severe FAMACHA© scores, particularly when the goal was to identify animals requiring closer attention.

Freitas et al. [12] evaluated deep CNN models on sheep ocular conjunctiva images using packed cell volume (PCV) as the reference label. Their best binary classifier, VGG19, achieved an F1-score of 0.62 and an anemia recall of 0.83 using PCV < 27% as the anemia threshold. Their task differs from ours because PCV is a laboratory-derived measurement, whereas FAMACHA© is a field-based visual score. Even so, their study provides an important benchmark for deep learning in ocular-conjunctival anemia screening. Our ViT-B/16 model achieved an F1-score of 0.879 and an anemia recall of 0.924, while the selected U-Net + ResNet-34 segmentation model achieved a higher IoU than their reported conjunctiva segmentation result. These differences should be interpreted with caution due to differences in species, label definitions, and annotation procedures.

The test set in this study was intentionally enriched for anemic cases and therefore does not represent the prevalence expected in a typical field population. To examine the effect of lower anemia prevalence, we estimated positive predictive value (PPV) from the sensitivity and specificity observed on the held-out test set using

(1) $$\mathrm{PPV}=\frac{\mathrm{Sensitivity}\times \mathrm{Prevalence}}{\mathrm{Sensitivity}\times \mathrm{Prevalence}+\left(1-\mathrm{Specificity}\right)\left(1-\mathrm{Prevalence}\right)}$$

Assuming anemia prevalences of 5%, 10%, and 15%, the estimated PPV for ViT-B/16 was 18.4%, 32.3%, and 43.1%, respectively. For MobileNetV4-Small, which had the highest specificity, the corresponding PPVs were 20.7%, 35.5%, and 46.6%. At an assumed prevalence of 5%, these operating characteristics correspond to approximately 204 false-positive classifications per 1000 screened animals for ViT-B/16 and 166 for MobileNetV4-Small. These estimates illustrate that PPV decreases substantially when anemia is uncommon, even when sensitivity remains high. These values are prevalence-based projections rather than direct field-validation results. Therefore, the proposed system should be interpreted as a screening tool rather than a stand-alone treatment decision system, and its performance should be prospectively evaluated in goat populations with naturally occurring anemia prevalence.

Overall, the main distinction of this work lies in the evaluation setting rather than in the best point estimate alone. The dataset was goat-specific; the split prevents the same animal from appearing across the training, validation, and test subsets; and the model comparison includes uncertainty estimates, paired statistical testing, and deployment-oriented analysis. These elements make the results more informative for practical screening than accuracy alone.

### 5.2. On-Device Latency

The on-device benchmark showed that computational cost depends on both segmentation and classification. Classification alone varied widely across the six backbones, from 3.99 milliseconds (ms) for MobileNetV4-Small to 642.29 ms for ViT-B/16. However, when the separately measured segmentation and classification latencies were combined, the U-Net segmentation stage became the dominant source of inference time for the lightweight classifiers.

Among the three U-Net segmentation backbones, ResNet-34 provided the best practical balance. It achieved the highest segmentation IoU in Section 4.1 and required 475.05 ± 18.17 ms per inference on the Samsung SM-A556E. ResNet-18 was faster at 351.94 ± 43.92 ms, but had slightly lower segmentation performance, while ResNet-50 was much slower at 3789.79 ± 530.36 ms. For this reason, we used a U-Net with a ResNet-34 encoder as the segmentation stage for the combined two-stage latency analysis. Table 11 summarizes the on-device latency and segmentation performance of the three U-Net encoder configurations.

Table 12 summarizes the estimated combined two-stage inference latency obtained by summing the separately measured U-Net-ResNet34 segmentation and classification latencies. The estimated combined latency ranged from 479.04 ms to 1117.34 ms. The lowest estimate was obtained with U-Net-ResNet34 and MobileNetV4-Small at 479.04 ms. EfficientNet-B0 and MobileViT-v2-1.0 produced estimates of 493.33 ms and 517.59 ms, respectively. ViT-B/16 remained the strongest recall-oriented classifier, but its estimated combined latency exceeded 1 s because of its higher classification inference cost.

These results demonstrate on-device inference feasibility under the tested smartphone configuration. For lightweight classifiers, most of the estimated combined inference time comes from the segmentation model rather than the classifier. Therefore, future optimization should focus not only on replacing the classification backbone, but also on accelerating the segmentation stage through lower input resolution, model compression, pruning, or quantization. The fastest configuration had an estimated combined model-inference latency of approximately 0.48 s per image on the tested device. This estimate excludes the preprocessing and intermediate operations described in Section 3.8 and therefore does not represent the runtime of the complete application.

## 6. Conclusions

This study presented an animal-level deep learning framework for FAMACHA©-based anemia screening using ocular conjunctival images. The pipeline combines U-Net-based conjunctival segmentation with deep image classification and was evaluated on 1995 FAMACHA©-labeled images from 141 goats collected across three acquisition campaigns. The FAMACHA© score was found to be strongly positive and connected with fecal egg counts (r = 0.7082, R^2^ = 0.5015, *p* < 0.0001), indicating a strong correlation between the FEC method and FAMACHA©.

The selected U-Net + ResNet-34 segmentation achieved a foreground IoU of 0.7342 and a Dice coefficient of 0.8372. For classification, ViT-B/16 achieved the strongest held-out test performance, with 0.861 accuracy, 0.879 anemia-class F1-score, and 0.924 anemia recall. MobileNetV4-Small performed close to ViT-B/16 while offering much lower smartphone inference latency, making it the most practical accuracy–latency option among the evaluated backbones. Overall, the results show that model choice for FAMACHA©-based screening should consider not only accuracy, but also anemia recall, specificity, statistical reliability, and computational cost.

Some limitations still persist. The dataset was collected using farms in one geographic area, so external validation across farm types, breeds, and management conditions is needed. Trained professionals provided the FAMACHA© scores, but inter-rater agreement was not assessed. Another limitation of the merged dataset is the inclusion of an anemia-enriched cohort; therefore, its class distribution should not be interpreted as reflecting natural herd-level prevalence. The segmentation model was trained and evaluated exclusively on Cohort 3 images, which were acquired under standardized tablet imaging. Although the resulting segmentation was applied across all cohorts in the integrated pipeline, its generalization to the imaging conditions of Cohorts 1 and 2 was not directly evaluated. Future work should extend polygon annotation to all cohorts to assess and improve cross-cohort segmentation robustness.

Although the held-out classification results incorporate automatically generated segmentation and cropping, the isolated contribution of segmentation errors to downstream classification performance was not quantified. Finally, the reported combined latency was estimated from separately measured segmentation and classification inference times and did not include image resizing, mask post-processing, bounding-box computation and cropping, image normalization, animal handling, conjunctival exposure, image capture, data transfer, user-interface overhead, or complete application-level workflow timing.

An end-to-end mobile application integrating image acquisition, segmentation, cropping, classification, and final screening output has been developed. Future work will prospectively validate the complete application under realistic field conditions and quantify error propagation across the full pipeline. Additional evaluation should include independent goat populations, multi-evaluator FAMACHA© scoring, and paired PCV measurements. Future work should also include cohort-stratified segmentation evaluation across different cameras, lighting conditions, and acquisition settings. These steps would help move the proposed framework toward practical use in refugia-aware anthelmintic management.

## Figures and Tables

**Table 1:** Animal-level stratified split for the classification task. No goat appears in more than one subset.

Split	Images	Goats	Anemic	Non-Anemic	Anemic (%)
Train	1150	84	517	633	45.0
Validation	341	28	127	214	37.2
Test	504	29	276	228	54.8
Total	1995	141	920	1075	46.1

**Table 2:** Hyperparameter search/tuning selection range.

Hyperparameter	Search Range
Head learning rate	{3 × 10^−4^, 5 × 10^−4^, 1 × 10^−3^, 1.5 × 10^−3^, 2 × 10^−3^, 3 × 10^−3^}
Backbone learning rate	{1 × 10^−5^, 2 × 10^−5^, 5 × 10^−5^, 7 × 10^−5^, 1 × 10^−4^, 3 × 10^−4^, 5 × 10^−4^}
Weight decay	{1 × 10^−4^, 5 × 10^−4^, 1 × 10^−3^, 1 × 10^−2^, 5 × 10^−2^, 1 × 10^−1^}
Label smoothing	{0.0, 0.1}
Mixup α	{0.0, 0.2}
Optimizer	AdamW
Scheduler	Linear warmup + cosine decay
Head-only training epochs	1
LR warm-up epochs	2
Maximum epochs	30 (minimum 12 before early stop)

**Table 3:** Final per-model hyperparameters used during retraining and test-time evaluation.

Model	Head LR	Backbone LR	LR Strategy	Weight Decay	Drop_Rate	Drop_Path_Rate	Threshold
ResNet-50	1 × 10^−3^	3 × 10^−4^	Discriminative	1 × 10^−4^	0	0	0.4
ViT-B/16	1 × 10^−3^	5 × 10^−5^	Discriminative	1 × 10^−2^	0	0	0.7
Swin-T	1 × 10^−4^	1 × 10^−4^	Uniform	5 × 10^−2^	0.1	0.2	0.5
EfficientNet-B0	1 × 10^−3^	1 × 10^−3^	Uniform	1 × 10^−4^	0.2	0.2	0.5
MobileNetV4-Small	5 × 10^−4^	1 × 10^−4^	Discriminative	5 × 10^−4^	0.1	0.05	0.45
MobileViT-v2-1.0	1 × 10^−3^	3 × 10^−4^	Discriminative	1 × 10^−4^	0.1	0.05	0.35

Note: Head LR: Head Learning Rate, Backbone LR: Backbone Learning Rate, and LR Strategy: Learning Rate Strategy.

**Table 4:** Result of U-Net Segmentation Based on ResNet18, ResNet34, and ResNet50.

Encoder	IoU	Dice	Precision	Recall	Pixel Acc.
ResNet-18	0.7291 [0.7147, 0.7430]	0.8334 [0.8198, 0.8458]	0.8455	0.8506	0.9993
ResNet-34	0.7342 [0.7199, 0.7479]	0.8372 [0.8236, 0.8494]	0.8499	0.8517	0.9994
ResNet-50	0.7286 [0.7146, 0.7422]	0.8330 [0.8192, 0.8457]	0.8739	0.8204	0.9994

**Table 5:** Nested cross-validation results across five outer folds. Values are reported as mean ± standard deviation.

Model	Accuracy (%)	F1 Anemia (%)
Swin-T	86.92 ± 5.05	84.86 ± 5.41
MobileViT-v2-1.0	86.52 ± 8.42	83.63 ± 9.74
ResNet-50	86.26 ± 4.58	84.29 ± 4.74
MobileNetV4-Small	86.18 ± 8.64	83.39 ± 8.10
EfficientNet-B0	84.74 ± 9.09	83.19 ± 8.25
ViT-B/16	83.23 ± 5.65	78.58 ± 8.35

**Table 6:** Per-fold outer accuracy (%) under the nested 5 × 3 cross-validation protocol.

Model	Fold 1	Fold 2	Fold 3	Fold 4	Fold 5	Mean	SD
Swin-T	89.86	81.00	88.25	82.49	93.00	86.92	5.05
MobileViT-v2-1.0	85.15	94.82	80.80	76.43	95.38	86.52	8.42
ResNet-50	90.54	79.33	86.91	84.51	90.00	86.26	4.58
MobileNetV4-Small	87.34	93.53	76.31	78.34	95.38	86.18	8.64
EfficientNet-B0	82.10	93.20	75.31	77.71	95.38	84.74	9.09
ViT-B/16	84.80	77.67	85.57	77.44	90.67	83.23	5.65

**Table 7:** Held-out test set performance (*n* = 504 images from 29 goats) for the six classification backbones at their validation-selected operating thresholds. Values in brackets are 95% confidence intervals from *B* = 4000 goat-level cluster bootstrap resamples.

Model	Threshold	Accuracy	F1 (Anemia)	Recall (Anemia)	Precision (Anemia)	Specificity	Balanced Accuracy
ViT-B/16	0.70	0.861 [0.779, 0.921]	0.879 [0.697, 0.941]	0.924 [0.753, 0.985]	0.839 [0.627, 0.928]	0.785 [0.683, 0.875]	0.855 [0.774, 0.909]
MobileNetV4-Small	0.45	0.847 [0.747, 0.908]	0.861 [0.642, 0.928]	0.866 [0.615, 0.954]	0.857 [0.635, 0.937]	0.825 [0.739, 0.899]	0.845 [0.729, 0.901]
EfficientNet-B0	0.50	0.825 [0.722, 0.890]	0.847 [0.625, 0.917]	0.880 [0.667, 0.964]	0.815 [0.566, 0.912]	0.759 [0.667, 0.844]	0.820 [0.713, 0.880]
ResNet-50	0.40	0.823 [0.729, 0.887]	0.846 [0.623, 0.918]	0.884 [0.685, 0.955]	0.811 [0.543, 0.913]	0.750 [0.674, 0.833]	0.817 [0.722, 0.870]
Swin-T	0.50	0.819 [0.705, 0.899]	0.847 [0.611, 0.927]	0.909 [0.679, 0.987]	0.792 [0.521, 0.902]	0.711 [0.611, 0.816]	0.810 [0.698, 0.876]
MobileViT-v2-1.0	0.35	0.802 [0.680, 0.884]	0.829 [0.563, 0.916]	0.877 [0.614, 0.967]	0.786 [0.495, 0.904]	0.711 [0.603, 0.820]	0.794 [0.672, 0.865]

**Table 8:** Cohort-stratified classification performance on the held-out test set using the validation-selected operating threshold for each model. Cohorts 1&2 included 131 images from 17 goats, whereas Cohort 3 included 373 images from 12 goats.

Model	Cohort 1&2			Cohort 3		
	Acc.	Recall	Bal. Acc.	Acc.	Recall	Bal. Acc.
ResNet-50	0.786	0.636	0.726	0.836	0.906	0.797
ViT-B/16	0.824	0.773	0.804	0.874	0.937	0.838
Swin-T	0.802	0.727	0.772	0.826	0.925	0.769
EfficientNet-B0	0.786	0.773	0.781	0.839	0.890	0.810
MobileNetV4-Small	0.832	0.636	0.754	0.853	0.886	0.834
MobileViT-v2-1.0	0.786	0.545	0.690	0.807	0.906	0.751

**Table 9:** Pairwise goat-block permutation tests on the held-out test set (504 images from 29 goats). Raw *p*-values were adjusted using Holm’s procedure for 15 comparisons. Significance is based on Holm-adjusted *p*-values: * *p* < 0.05; ns = not significant.

Model A	Model B	Accuracy Difference (A − B)	Raw *p*-Value	Holm-Adjusted *p*-Value	Sig.
ResNet-50	ViT-B/16	−0.0377	0.0727	0.8724	ns
ResNet-50	Swin-T	0.004	0.9281	1.0000	ns
ResNet-50	EfficientNet-B0	−0.002	1.0000	1.0000	ns
ResNet-50	MobileNetV4-Small	−0.0238	0.3635	1.0000	ns
ResNet-50	MobileViT-v2-1.0	0.0218	0.3101	1.0000	ns
ViT-B/16	Swin-T	0.0417	0.0347	0.4858	ns
ViT-B/16	EfficientNet-B0	0.0357	0.1181	1.0000	ns
ViT-B/16	MobileNetV4-Small	0.0139	0.5178	1.0000	ns
ViT-B/16	MobileViT-v2-1.0	0.0595	0.0017	0.0255	*
Swin-T	EfficientNet-B0	−0.006	0.8829	1.0000	ns
Swin-T	MobileNetV4-Small	−0.0278	0.2694	1.0000	ns
Swin-T	MobileViT-v2-1.0	0.0179	0.3924	1.0000	ns
EfficientNet-B0	MobileNetV4-Small	−0.0218	0.2286	1.0000	ns
EfficientNet-B0	MobileViT-v2-1.0	0.0238	0.4379	1.0000	ns
MobileNetV4-Small	MobileViT-v2-1.0	0.0456	0.0486	0.6318	ns

**Table 10:** Comparison with prior image-based anemia screening studies in small ruminants. Recall and F1 refer to the anemia-positive class where applicable. Task formulations differ across studies, so do not interpret the values as direct head-to-head comparisons.

Study	Species	Dataset Size	Anemia Definition	Best Model	F1	Recall
de Souza et al. [11]	Sheep	317 img	FAMACHA© 3-5	SVM (RGB)	0.88	0.90
Freitas et al. [3]	Sheep	1156 img	FAMACHA© (5-class)	Random Forest	—	0.81 (F3)
Freitas et al. [12]	Sheep	3441 img	PCV < 27%	VGG19	0.62	0.83
This work (2026)	Goat	1995 img	FAMACHA© 4-5	ViT-B/16	0.879	0.924

**Table 11:** On-device segmentation-stage latency measured on a Samsung SM-A556E smartphone using TensorFlow Lite under the tested inference configuration.

U-Net Backbone	Parameters (M)	MACs (G)	Model Size (MB)	Latency (ms)	Seg. IoU
ResNet-18	~14	22.85	55	351.94 ± 43.92	0.7291
ResNet-34	~24	32.52	93	475.05 ± 18.17	0.7342
ResNet-50	~33	123.15	274	3789.79 ± 530.36	0.7286

**Table 12:** Estimated combined two-stage inference latency using U-Net-ResNet34 segmentation followed by each classification backbone. Total latency was estimated as the sum of the separately measured segmentation and classification inference times.

Pipeline	Segmentation (ms)	Classification (ms)	Estimated Total (ms)	Approx. Throughput
U-Net-R34 + MobileNetV4-Small	475.05	3.99	479.04	~2.1 inf/s
U-Net-R34 + EfficientNet-B0	475.05	18.28	493.33	~2.0 inf/s
U-Net-R34 + MobileViT-v2-1.0	475.05	42.54	517.59	~1.9 inf/s
U-Net-R34 + ResNet-50	475.05	78.76	553.81	~1.8 inf/s
U-Net-R34 + Swin-T	475.05	172.35	647.40	~1.5 inf/s
U-Net-R34 + ViT-B/16	475.05	642.29	1117.34	~0.9 inf/s

## Data Availability

The data used for this study are available from the corresponding author upon request. The data that supports the findings of this research are not publicly available due to ongoing intellectual property protection considerations.
